# Neural Mechanisms of Dorsal and Ventral Visual Regions during Text Reading

**DOI:** 10.3389/fpsyg.2016.01399

**Published:** 2016-09-15

**Authors:** Wei Zhou, Xiaojuan Wang, Zhichao Xia, Yanchao Bi, Ping Li, Hua Shu

**Affiliations:** ^1^Beijing Key Lab of Learning and Cognition, Department of Psychology, Capital Normal UniversityBeijing, China; ^2^Beijing Advanced Innovation Center for Imaging Technology, Capital Normal UniversityBeijing, China; ^3^State Key Laboratory of Cognitive Neuroscience and Learning and IDG/McGovern Institute for Brain Research, Beijing Normal UniversityBeijing, China; ^4^School of Psychology, Shaanxi Normal UniversityXi’an, China; ^5^Department of Psychology and Center for Brain, Behavior and Cognition, Pennsylvania State University, University ParkPA, USA

**Keywords:** text reading, dorsal visual region, ventral visual region, functional connectivity, effective connectivity

## Abstract

When reading a narrative text, both the dorsal and ventral visual systems are activated. To illustrate the patterns of interactions between the dorsal and ventral visual systems in text reading, we conducted analyses of functional connectivity (FC) and effective connectivity (EC) in a left-hemispheric network for reading-driven functional magnetic resonance imaging (fMRI) and resting-state fMRI (rs-fMRI) data. In reading-driven fMRI (Experiment 1), we found significant FCs among the left middle frontal gyrus (MFG), the left intraparietal sulcus (IPS), and the visual word form area (VWFA), and there were top–down effects from the left MFG to the left IPS, from the left MFG to the VWFA, and from the left IPS to the VWFA. In rs-fMRI (Experiment 2), we identified FCs and ECs for MFG-IPS and IPS-VWFA connections. In addition, the brain–behavior relationship in resting states showed that the dorsal connection was more associated with reading fluency relative to lexical decision. The combination of two experiments revealed that the MFG-IPS and the VWFA-IPS connections were shared connections both in reading-driven fMRI and rs-fMRI, and that the MFG-VWFA was specific connectivity in reading-driven fMRI. These results suggest that top–down effects from the dorsal visual system to ventral visual system play an important role in text reading.

## Introduction

Reading of a narrative text (henceforth ‘text reading’) plays a key role in our daily lives. Text reading requires a dynamic integration of vision, visual attention, and linguistic processes of the materials. When reading words, the ventral visual stream is activated which involves the visual word form area (VWFA) as a central hub (Cohen et al., 2000, 2002; Cohen and Dehaene, 2004). According to the dual-route theory of visual processing (e.g., Goodale and Milner, 1992), the division of labor between a dorsal “where” stream and a ventral “what” stream is one of the most fundamental principles of information processing in the brain (Ungerleider and Haxby, 1994). Similarly, the processes involved in text reading may also follow this dual-route principle. While previous functional magnetic resonance imaging (fMRI) studies have generally implicated a key role of the ventral visual system for word reading (for a review, see Dehaene and Cohen, 2011), recent fMRI studies in naturalistic text reading have proposed that the dorsal visual regions [e.g., intraparietal sulcus (IPS)] play a role in visual/spatial attention or eye movements during reading of an entire sentence (Hillen et al., 2013; Choi et al., 2014). However, little is known about how the dorsal visual system interacts with the ventral visual region during text reading. The present study was specifically aimed at addressing this question.

Brain connectivity analyses, including both functional connectivity (FC) and effective connectivity (EC), have become important for understanding the inter-regional associations in both task-driven fMRI and resting-state fMRI (rs-fMRI; see Friston, 2011 for a review). Such analyses in reading research have revealed the dynamics of cooperation and causal influence among several key language regions including the left perisylvian cortex, as revealed by task-driven fMRI studies of word reading (e.g., Booth et al., 2008; Richardson et al., 2011; Duncan et al., 2013; Kwok et al., 2015) and rs-fMRI studies (e.g., Hampson et al., 2002, 2006; Koyama et al., 2010; Li et al., 2013; Schurz et al., 2014a). Interestingly, recent rs-fMRI studies have consistently shown that the VWFA was functionally connected to dorsal attention regions (Vogel et al., 2011; Wang X.S. et al., 2015). In a rs-fMRI study with children, Zhou et al. (2015) have investigated the FC seed maps of two central nodes for the dorsal and ventral visual streams respectively (i.e., VWFA and IPS), and found that FCs from the VWFA and the left IPS were both connected to the left middle frontal gyrus (MFG). The left MFG was proposed as a convergent region for the dorsal and ventral visual systems during text reading. They also demonstrated that the FC strength between IPS-MFG was positively correlated with the scores of reading fluency but not the scores of lexical decision.

While task-driven fMRI provides an opportunity to study the evoked neural mechanism of cognition (e.g., reading), it is likely influenced by task-induced factors (Friston, 2005; Koyama et al., 2010). In contrast, the rs-fMRI, which measures spontaneously activation during rest, can reveal the brain’s intrinsic functional organization that relates to cognition. Therefore, the combination of task-driven fMRI and rs-fMRI provides a good approach to investigate the fundamental and reliable neural mechanism of cognition (Kannurpatti et al., 2012; Rosazza et al., 2014). However, this approach has been rarely used in the field of reading. It is unknown based on our previous studies whether the above connectivity findings in children could be applied to adults, and whether the findings in rs-fMRI could be generalized to reading-driven fMRI. In order to address these questions, we conducted the FC analyses in a reading-driven fMRI and a rs-fMRI experiments with adults. Although, a number of regions in the perisylvian cortex may also be involved in text reading (e.g., see Price, 2012 for a comprehensive review), the present study mainly focused on the FCs for the dorsal and ventral visual streams respectively (see also Zhou et al., 2015). It has been suggested that there is a common mechanism between task-driven fMRI and rs-fMRI (Mennes et al., 2010), but weak correspondence may exist for regions whose patterns of evoked functional interactions are more adaptive and context-dependent (Mennes et al., 2013; see also Sporns, 2011). Thus, it is important to carry out similar FC analyses in both task-driven fMRI and rs-fMRI, so that we can identify whether the brain connections among the dorsal and ventral visual systems are stable or context-dependent, in text reading and other similar processes.

A further important aim of the current study was to ask how the regions in the dorsal and ventral visual systems causally affect each other in text reading. The directional influence (i.e., top–down versus bottom–up) between the dorsal and ventral visual streams is a hotly debated issue in the discussion of the dual-route mechanisms for visual attention tasks. While the automatic “bottom–up” capture of attention is driven by stimulus properties derived from the ventral visual system, the volitional “top–down” modulation is due to selective visual attention and knowledge about the current task situated in the dorsal visual system (Kastner and Ungerleider, 2000; Buschman and Miller, 2007). As a specific visual-related task, text reading should also require such a dynamically interactive system: when word presentation in the ventral visual system initiates a bottom–up effect (Schurz et al., 2014a), high level factors situated in the dorsal system (e.g., visual attention or situation construction; Yarkoni et al., 2008; Vidyasagar and Pammer, 2010) are likely to exert a top–down control. Compared to the tasks using isolated word presentation paradigms (e.g., lexical decision, semantic categorization), the text reading tasks (e.g., reading fluency) are more likely to be dominated by the top–down control process (Radach et al., 2008). In order to test the existence of bidirectional influences (i.e., top–down versus bottom–up) between the dorsal and ventral visual systems in text reading, we employed Granger causality analysis (GCA) for EC analyses, which has been frequently used in previous task-driven fMRI and rs-fMRI studies (Roebroeck et al., 2005; Seth et al., 2015). While non-directional FC reveals the synchronization between brain regions, directional EC reflects the influence from one brain region to another. The use of both kinds of analysis not only verifies specific coherent systems in the brain but also uncovers the dynamic nature of these systems.

Although, the extant literature has pointed to interactions between the dorsal and ventral visual streams in text reading, the detailed picture of such interaction has not emerged. In this study, we hypothesized that (a) there are significant FCs and ECs among regions in the dorsal and ventral visual systems in reading-driven fMRI, (b) the brain connections in rs-fMRI are generally similar to those in reading-driven fMRI, as the resting-state connectivity is assumed to reflect a history of consistent and repeated co-activations of areas (Dosenbach et al., 2007; Fair et al., 2007), and (c) the dorsal visual connection is more correlated with text reading relative to single word processing. To test these hypotheses, we carried out two fMRI experiments for FC and EC analyses: on the basis of existing data from Wang X.J. et al. (2015), we selected regions of interest (ROIs) as two central nodes (i.e., IPS and VWFA) in the dorsal and ventral visual systems and one convergence node (i.e., MFG) for these two systems in the frontal cortex. Wang X.J. et al. (2015) conducted analyses of whole-brain and ROI-based activations in naturalistic text reading of both English and Chinese. In the current analyses in our Experiment 1, we examined only the dataset from Chinese text reading to identify connectivity patterns so as to compare with rs-fMRI data from Experiment 2. In Experiment 2, the same ROIs as in Experiment 1 were used for FC and EC analyses in a set of data from the participants’ rs-fMRI. Finally, in order to distinguish the functions of the dorsal and ventral visual connections and to identify brain-behavior relations, we calculated correlations between the strength of brain connections in rs-fMRI and the behavioral reading scores that were based on fluent reading against lexical decision tasks. As fluent reading engages more cognitive resources such as visual attention allocation (Rayner, 2009) relative to single word processing, we expected different brain patterns for these two tasks, especially in dorsal attention regions.

## Experiment 1

### Methods

#### Participants

Sixteen undergraduate or graduate students (age *mean* = 22 years, *standard deviation* = ± 1.7; eight females and eight males) from Beijing Normal University participated in the experiment. All were native speakers of Chinese with normal or corrected-to-normal vision, and were right-handed according to a translated version of handedness assessment instrument (Oldfield, 1971). No participants had a history of neurological diseases or psychiatric disorders. All the participants signed informed written consent before the experiment. The study was approved by the local ethics committee at the Beijing Normal University’s Imaging Center for Brain Research.

#### Stimuli and Procedure

Six fairytale stories (written by Hans Christian Andersen originally and translated by Wang X.J. et al., 2015 into Chinese) were selected as the materials for naturalistic reading. Each sentence of the story contained an average of 12 Chinese characters, and was presented entirely on the screen. The current analysis was based on the data of visual story reading reported in Wang X.J. et al. (2015). Participants silently read stories presented sentence by sentence, and also completed in the scanner a set of four-multiple-choice comprehension questions after each story. All participants’ accuracies for comprehension questions were above 90%, indicating that they understood the narratives quite well. Full details concerning the stimuli and procedure can be found in the description of the Materials and Procedure of Experiment 1 in Wang X.J. et al. (2015).

#### Imaging Acquisitions

Magnetic resonance imaging (MRI) data were obtained on a SIEMENS TRIO 3-Tesla scanner in the Beijing Normal University’s Imaging Center for Brain Research. We collected fMRI data using an echo-planar imaging (EPI) sequence with the following parameters: axial slices = 41, thickness = 3.0mm, in-plane resolution = 64 × 64, TR = 2500 ms, TE = 30 ms, flip angle = 90°, FOV = 200 mm × 200 mm, and voxel size = 3.125 mm × 3.125 mm × 3 mm.

#### Data Analyses

##### Data preprocessing

We used *SPM8*^1^ for all data processing and analyses. During activation analyses in *SPM8*, the data were first motion corrected and the images were then normalized using a standard EPI template based on the Montreal Neurological Institute (MNI) reference brain. Then functional volumes were resampled to isotropic 3 mm^3^ voxels and spatially smoothed with a 6 mm full width at half maximum isotropic Gaussian kernel. With respect to FC and EC analyses, additional preprocessing was conducted with *REST*, a MATLAB-based toolkit that interfaces with *SPM8* (Song et al., 2011^2^), as follows: for each passive reading block in each run, the first four volumes (10 s) were discarded to minimize the effects of hemodynamic delay from previous conditions. After linear trend removal, several nuisance variables, including six head-motion parameters, the averaged signal from white matter and ventricles, and the global signal (averaged BOLD signals across all voxel) were removed by multiple linear regression analysis. As recent studies have argued that the regression of global signals will distort FC measures (e.g., Saad et al., 2012), we also explored whether global signal removal would affect the results but found no significant differences with or without global signal removal (see Supplementary Materials).

##### Activation analyses

Images were first assigned to cross fixation or task blocks. For each participant, a *t*-test was performed at each voxel comparing signal level during reading blocks to signal level during fixation cross blocks, yielding contrast maps of activation for each participant. Group analysis including all contrast maps of participants was performed using one-sample *t*-test (FDR corrected, *p* < 0.05, *k* > 50) to show common regions of activation involved in text reading. Because our current study is focused on the functional networks of the dorsal and ventral visual regions and the relationship between them, we selected two representative ROIs for dorsal (i.e., left IPS) and ventral (i.e., VWFA) visual pathways from the peak activation coordinates.

##### FC analyses

Functional connectivity analyses were based on *REST-Fun.connectivity*, a plug-in implemented in the *REST* software (Song et al., 2011^2^). For each participant, the time course was extracted for 6 mm spheres centered on the left IPS and the VWFA. Because brain activation associated with text reading was left lateralized, so we mainly focused on brain connections in the left hemisphere. The results for brain connections in the right hemisphere were provided in Supplementary Material. The regional time course was calculated by averaging the time series of all of the voxels within the seed region. Then, the time course for each of the seed regions was correlated with every other voxel in the brain to generate individual seed maps (Fisher-*r*-to-*z* transformed). Finally, for each seed region, group-level analyses were performed using one-sample *t*-test (FDR corrected, *p* < 0.05, *k* > 50) for the left IPS and the VWFA seed maps respectively. Then these two seed maps were overlaid on one another. Given the importance of the prefrontal cortex shown in previous work, we added the central coordinate of the prefrontal region (i.e., left MFG) in the subsequent analyses. In addition, we calculated the strength of FCs among the MFG, IPS, and VWFA to perform confirmatory analyses by using one-sample *t*-test. Following the conventions for each type of analysis, we used correlation coefficient Z*r* (Fisher-*r*-to-*z* transformed) for the strength of FCs. The results could then be visualized using the template surface of smoothed ICBM152 in *BrainNet Viewer* (Xia et al., 2013).

##### EC analyses

Granger causality analysis is an EC method that quantifies the improvement in predicting one brain region’s signal that results from inclusion of another region’s signal (Granger, 1969). GCA was first proposed for determining whether the past value of a time course could correctly forecast the current value of another by using vector autoregressive models (Ding et al., 2000). If the current value of time course Y could be more accurately estimated by the combination of past value of time courses X and Y than the past value of Y alone, then X has Granger causal influence on Y (Roebroeck et al., 2005; Bressler and Seth, 2011). In this study, coefficient-based GCA used the regression coefficient β in vector autoregressive models to estimate Granger influence. A positive value of β may indicate positive influence, and a negative β may indicate inhibitory influence (Palaniyappan et al., 2013). Based on the results of activation analyses and FC analyses, the left MFG, left IPS, and VWFA were selected as sphere seeds with a radius of 6 mm for the GCA. The individual ROI-wise signed-path coefficient GCA was performed using *REST-GCA*. Then the β coefficients for the group were tested by one-sample *t*-test. The results were visualized using the template surface of smoothed ICBM152 in *BrainNet Viewer* (Xia et al., 2013).

### Results

#### Brain Activation in Text Reading

**FIGURE 1:**
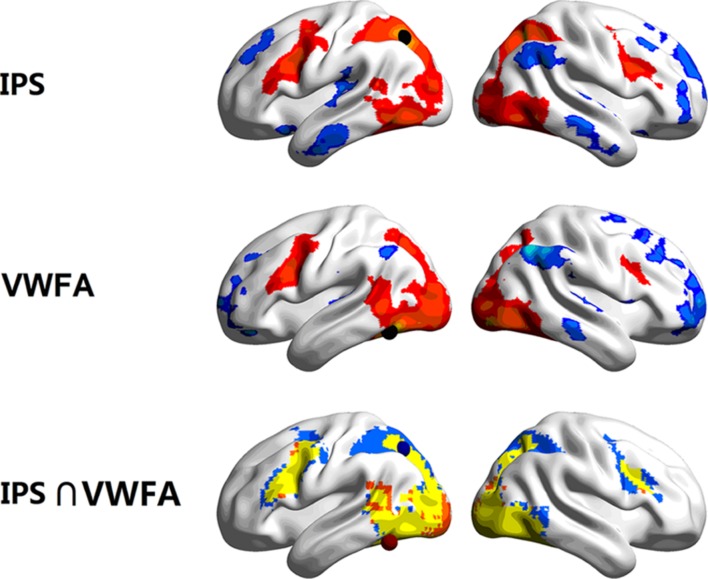
**Task-driven functional connectivity (FC) seed maps of the left IPS (**top**) and the VWFA (**middle**), and the map that has both IPS and VWFA seed regions (**bottom**, blue for the left IPS, orange for the VWFA, and yellow for the overlap in activation).** Maps displays voxels showing significant correlations with the time courses of seed regions (FDR corrected, *p* < 0.05, *k* > 50). The locations of the seeds (*r* = 6 mm) are marked with spheres.

As reported in the study of Wang and colleagues (see the top panel results for Chinese reading in Figure 1C of Wang X.J. et al., 2015), reading associated activation patterns included posterior clusters encompassing the bilateral occipital lobe and fusiform gyrus (including the VWFA), temporal clusters encompassing the bilateral middle temporal gyrus (MTG) and temporal pole (TP), frontal clusters encompassing the bilateral MFG and inferior frontal gyrus (IFG), and other distributed clusters in the bilateral superior frontal gyrus (SFG), left angular gyrus (AG), and left IPS. These results from Chinese text reading were consistent with the brain activation patterns shown for normal text reading as reported in Choi et al. (2014) for English. Based on these activation patterns and goals of the current study, two representative regions were selected from peak coordinates situated in the dorsal and ventral visual streams for the FC and EC analyses: the left IPS (MNI: -24, -66, 48) and the VWFA (MNI: -42, -57, -15).

#### FC in Text Reading

The time course for each of the seed regions (i.e., left IPS and VWFA) was correlated with every other voxel in the brain to generate individual seed maps. Then the group-level seed maps were computed for statistical significance by using one-sample *t*-test (FDR corrected, *p* < 0.05, *k* > 50). The seed map of the left IPS revealed a network composed of the bilateral VWFA, IPS, FEF (frontal eye field), MFG, and left MTG. The seed map of the VWFA was very similar to that of the left IPS, as shown in **Figure 1**. As the regions which had negative FCs with the left IPS and the VWFA may belong to a different functional system, the following analyses mainly focused on positively correlated regions within the dorsal-ventral visual network.

**FIGURE 2:**
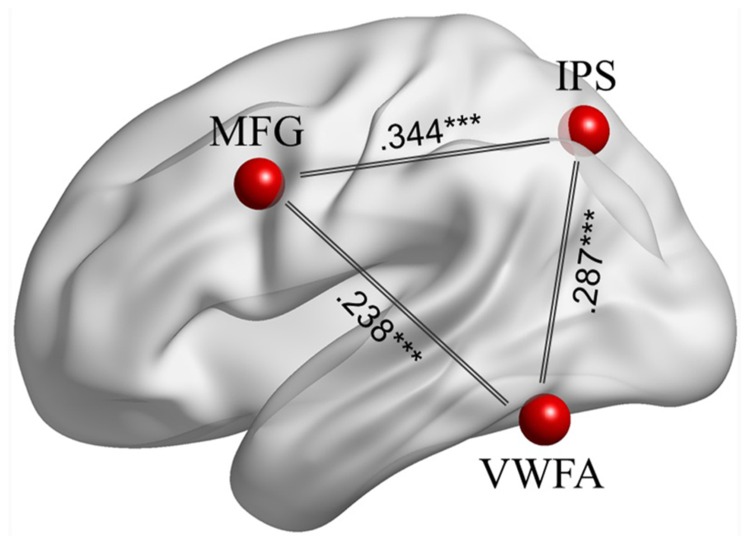
**The FCs among the left MFG, left IPS, and VWFA during task-driven fMRI.** All pair-wise correlations are significant at ^∗∗∗^*p* < 0.001.

After overlaying the seed maps of the left IPS and the VWFA, we observed that the MFG (**Figure 1**) was functionally connected to both the left IPS and the VWFA. Then the time course was extracted based on a 6 mm sphere centered on the central coordinate (MNI: -42, 9, 36) of the MFG. A ROI-wise manner calculation showed that the time courses among the left MFG, left IPS and VWFA were significantly correlated with each other (between MFG and IPS: *Z_r_* = 0.344, *p* < 0.001; between MFG and VWFA: *Z_r_* = 0.238, *p* < 0.001; between IPS and VWFA: *Z_r_* = 0.287, *p* < 0.001). **Figure 2** shows the FC connectivity patterns and correlations.

#### EC in Text Reading

**FIGURE 3:**
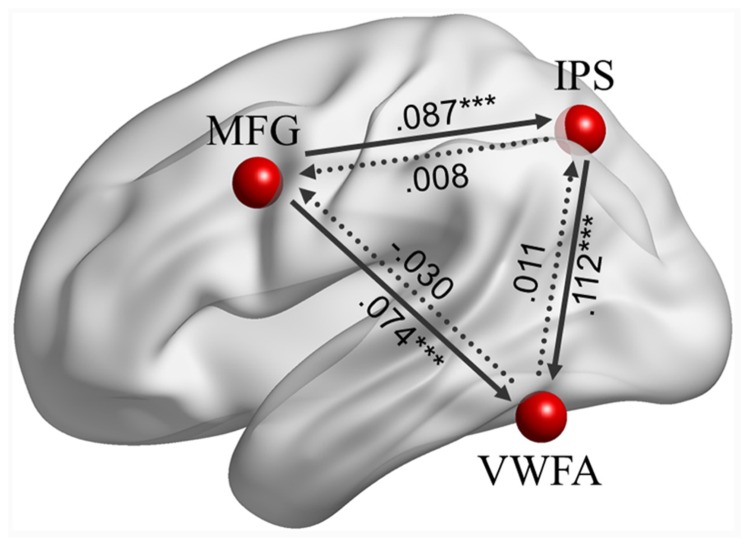
**The effective connectivity (ECs) among the left MFG, left IPS, and VWFA during task-driven fMRI.** Significant coefficients are at ^∗∗∗^*p* < 0.001, indicated by solid lines.

Effective connectivity analyses among the left MFG, left IPS and VWFA (see **Figure 3**) indicated that there were positive causal influences from the left MFG to the left IPS (β = 0.087, *p* < 0.001), from the left MFG to the VWFA (β = 0.074, *p* < 0.001), and from the left IPS to the VWFA (β = 0.112, *p* < 0.001). However, the ECs from the left IPS to the left MFG (β = 0.008, *p* = 0.775), from the VWFA to the left MFG (β = -0.030, *p* = 0.193), and from the VWFA to the left IPS (β = 0.011, *p* = 0.645) were not significant.

#### Summary

Text reading activated a large set of perceptual and language-related regions including the prefrontal cortex, the left IPS, and the VWFA, as reported in Wang X.J. et al. (2015). In our current analyses, we showed that the left MFG, left IPS, and VWFA were functionally connected with each other to form a network for text reading. In addition, we found significant top–down causal effects from the left MFG to both the VWFA and the left IPS, and from the left IPS to the VWFA. Bottom–up effects from VWFA to the other two areas, however, were not significant. To more closely understand the characteristics of the identified network, in Experiment 2 we conducted further analyses based on the rs-fMRI dataset as discussed earlier.

## Experiment 2

In Experiment 2, we conducted the FC and EC analyses with a new rs-fMRI dataset to identify similarities and differences in reading-driven fMRI versus rs-fMRI connectivity patterns. In addition, in order to understand how network connectivity patterns correlate with behaviors, we selected a reading fluency task and a lexical decision task because both tasks include visual aspects of reading, but are different in the degree of engagement in volitional attention control (Rayner, 2009).

### Methods

#### Participants

Twenty-eight undergraduate or graduate students (age *mean* = 22 years, *standard deviation* = ± 2.4; 14 females and 14 males) from Beijing Normal University participated in the experiment. No participants took part in Experiment 1. All were native speakers of Chinese with normal or corrected-to-normal vision, and were right-handed according to a translated version of handedness assessment instrument (Oldfield, 1971). No participants had a history of neurological diseases or psychiatric disorders. All the participants signed informed written consent before the experiment. The study was approved by the local ethics committee as in Experiment 1.

#### Behavioral Tasks

##### Reading fluency

This test was aimed at measuring efficiency in fluent reading. The materials included 100 sentences, increasing gradually in length from 6 to 158 characters for text reading, which the participants were required to complete. Participants were given 3 min to silently read as many sentences as possible and to indicate the correctness of the sentence meaning with ‘√’ or ‘×’ (also see Xue et al., 2013). The score of this task reflects the amount of characters that one can read per minute and was transformed to a z-score.

##### Lexical decision

This test could measure efficiency of orthographic processing for the single character. The materials included 200 items: 40 real characters (e.g., 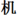), 40 pseudo-characters with real radicals in illegal positions (e.g., 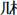), 40 non-characters with ill-formed components (e.g., 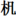), 40 scrambled strokes (e.g., 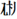) filled in 1 character space, and additional 40 real characters as fillers. Each stimulus was presented in the center of the computer screen for 1 s and participants were required to decide whether each stimulus was a real character or not (also see Su et al., 2015). Inverse efficiency score was calculated by dividing the response times by accuracy for the correct trials, which could provide a basis for processing efficiency independent of possible speed-accuracy trade-offs (Townsend and Ashby, 1978), and the score was transformed to an inverse number of the z-score.

#### Data Acquisitions

Magnetic resonance imaging data were similarly obtained on a SIEMENS TRIO 3-Tesla scanner as in Experiment 1. We collected rs-fMRI data using an EPI sequence with the following parameters: EPI functional volumes = 240, axial slices = 33, thickness = 4 mm, in-plane resolution = 64 × 64, TR = 2000 ms, TE = 30 ms, flip angle = 90°, FOV = 200 mm × 200 mm. During the resting-state session, the participants were instructed to remain motionless as best as they could and not to think actively about a particular idea with their eyes closed.

#### Data Analyses

##### Data preprocessing

Image preprocessing was carried out using the *DPARSF* (Yan and Zang, 2010), which is a batch tool for data preprocessing pipeline in *SPM8*. For each participant, after converting the DICOM files to NIFTI images, the first 10 time points were discarded to allow for scanner stabilization and the participant’s adaptation to the environment. The preprocessing on the remaining time points included: (1) slice timing for interleaved acquisitions, (2) a realigning step to correct for inter-scan head motions, (3) normalization of the functional images into the MNI space using an EPI template (Ashburner and Friston, 2000) and resampling to voxels of 3 mm × 3 mm × 3 mm in size, (4) spatial smoothing with a 4 mm FWHM Gaussian kernel, (5) removal of the trend of time courses, (6) temporal band-pass filtering (0.01–0.08 Hz), and (7) nuisance correction by regressing out six motion signals as well as individual white matter, cerebrospinal fluid and the global signals. We similarly explored the effect of global signal removing as in Experiment 1.

##### FC analyses

Functional connectivity analyses were based on *REST-Fun.connectivity*. We selected the same three ROIs (left MFG, left IPS, and VWFA) used in Experiment 1 for ROI-wise FC analyses. The time courses of ROIs were extracted and correlated with each other as in Experiment 1. In addition, we correlated the scores of reading fluency and lexical decision with the strength of significant FCs among the ROIs.

##### EC analyses

Effective connectivity analyses were based on *REST-GCA*. As in Experiment 1, we calculated the signed-path coefficients of the GCA to investigate the causal relations among the three ROIs, and correlated the scores of reading fluency and lexical decision with the coefficients of significant ECs among the ROIs.

### Results

#### FC at Rest

**FIGURE 4:**
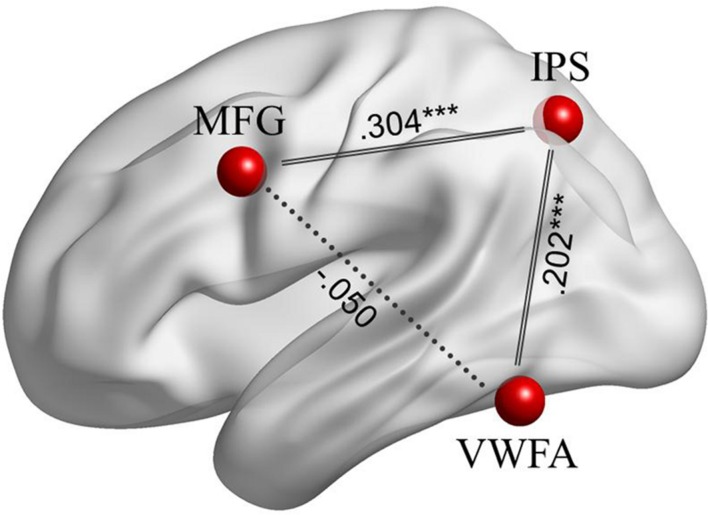
**The FCs among the left MFG, left IPS, and VWFA during resting-state fMRI.** Significant correlations are at ^∗∗∗^*p* < 0.001, indicated by solid lines.

**FIGURE 5:**
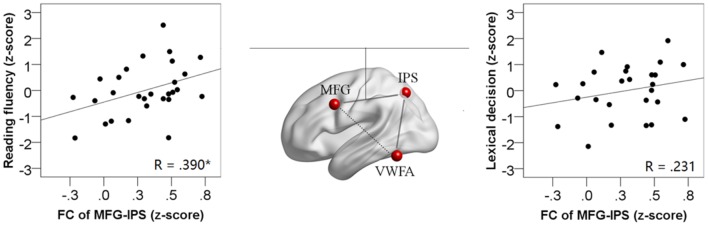
**The correlations between the FC strength of MFG-IPS (Fisher-*r*-to-*z* transformed) and the behavioral scores (the left scatterplot for reading fluency; the right scatterplot for lexical decision).** Significant correlations are at ^∗^*p* < 0.05.

In the ROI-wise analyses, as shown in **Figure 4**, there were significant and positive correlations of the time courses between the left MFG and the left IPS (*Z_r_* = 0.304, *p* < 0.001) and between the left IPS and the VWFA (*Z_r_* = 0.202, *p* < 0.001), but not between the left MFG and the VWFA (*Z_r_* = -0.050, *p* = 0.322). In addition, as shown in **Figure 5**, the strength of FC for MFG-IPS only increased significantly with the scores of reading fluency (*r* = 0.390, *p* = 0.040), but not with the scores of lexical decision (*r* = 0.231, *p* = 0.255). However, the strength of FC for VWFA-IPS did not significantly correlate with the scores of reading fluency (*r* = -0.332, *p* = 0.084), and weakly correlated with the scores of lexical decision (*r* = -0.391, *p* = 0.049).

#### EC at Rest

**FIGURE 6:**
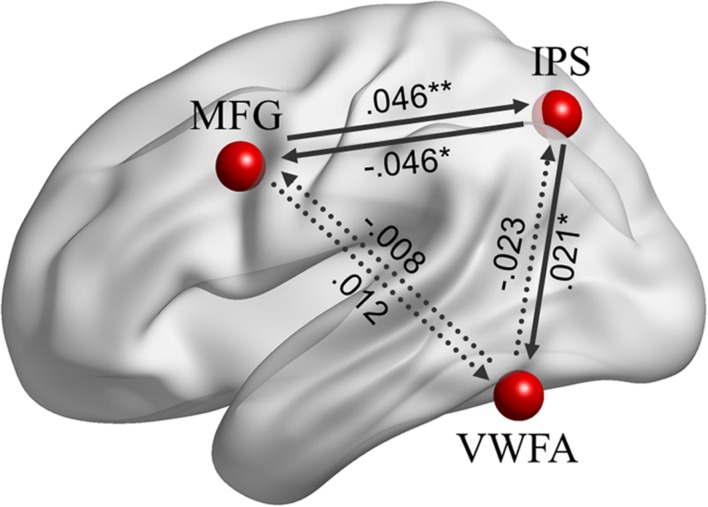
**The ECs among the left MFG, left IPS, and VWFA (*r* = 6 mm) during resting-state fMRI.** Significant coefficients are at ^∗∗^*p* < 0.01 and ^∗^*p* < 0.05.

Effective connectivity analyses among the left MFG, left IPS and VWFA, as shown in **Figure 6**, indicated that there were positive causal influences from the left MFG to the left IPS (β = 0.046, *p* = 0.003), from the left IPS to the VWFA (β = 0.021, *p* = 0.017), and negative causal influence from the left IPS to the left MFG (β = -0.046, *p* = 0.032). However, there was no significant EC from the left MFG to the VWFA (β = 0.012, *p* = 0.126), from the VWFA to the left MFG (β = -0.008, *p* = 0.794), or from the VWFA to the left IPS (β = -0.023, *p* = 0.304). Moreover, there was no brain–behavior correlation for the ECs with either lexical decision or reading fluency scores (*ps* > 0.05).

#### Summary

In Experiment 2 with rs-fMRI data, we identified similar FCs and ECs within the network of data from Experiment 1: there were significant FCs between the left MFG and the left IPS, and between the left IPS and the VWFA. There were significantly positive ECs from the left MFG to the left IPS, and from the left IPS to the VWFA, and a negative EC from the left IPS to the left MFG. However, we observed the lack of significant brain connectivity between the left MFG and the VWFA in resting states. In addition, we found that the FC strength for dorsal connection between MFG-IPS was significantly and positively correlated with the scores of reading fluency but not for lexical decision, and that the FC strength of IPS-VWFA was negatively correlated with the scores of lexical decision. These correlations are highly significant for our understanding of the role for the dorsal visual stream in text reading, as discussed below.

## General Discussion

Narrative text reading in our daily lives requires readers to integrate multiple words in an entire sentence, but traditional neuroimaging studies in reading have tended to present the stimuli as one word at a time in a rapid serial visual presentation paradigm (RSVP, see Price, 2012 for a review). Due to such methodological limitations, previous research has focused on the role of the ventral visual stream in text reading. More recently, researchers have started to work on paradigms that can present entire sentences or passages in fMRI experiments (e.g., Hillen et al., 2013; Regev et al., 2013; Choi et al., 2014; Schuster et al., 2015; Wang X.J. et al., 2015), and as a result the role of the dorsal visual regions in reading (Corbetta and Shulman, 2002; McDowell et al., 2008; Herweg et al., 2014) has now been examined. Although, the studies reviewed above have identified the distributed activations of both the dorsal and ventral visual regions in text reading, knowledge about the interactions between these two systems is lacking. To fill in this knowledge gap, the present study employed a dual-route approach for FC and EC analyses in task-driven fMRI and rs-fMRI. Although, there has been a “dual-route model of reading” concerned with a dorsal phonological route and a ventral orthographic route among left perisylvian regions (such as VWFA, IFG, inferior parietal lobe; see Coltheart et al., 2001; Jobard et al., 2003 for reviews), that model is focused on single-word reading rather than text reading and therefore it does not address research questions raised in the current study. Instead, our current study highlights a distinctive network that includes the VWFA and the dorsal visual attention regions (i.e., MFG and IPS). The findings of our reading-driven fMRI and rs-fMRI analyses converge to reveal the interactions between dorsal and ventral visual systems for text reading. As we expected, there were significant FCs and ECs among the left MFG, left IPS, and VWFA in both reading-driven fMRI and rs-fMRI.

In particular, the current study has highlighted directional influences among the dorsal and ventral visual regions by using EC analyses. The results have indicated a clear top–down modulation from the left MFG to the left IPS and from the left IPS to the VWFA in reading-driven fMRI and rs-fMRI. Most importantly, the current reading-driven fMRI has revealed dual top–down effects from the left MFG to both the dorsal region (i.e., left IPS) and the ventral region (i.e., left VWFA). These results were in agreement with the top–down effects from frontal-parietal regions to ventral visual regions or from frontal regions to parietal regions in vision research using GCA methods (e.g., Pessoa et al., 2003; Bressler et al., 2008; Hwang et al., 2010; Sneve et al., 2013). As previous studies have indicated, the MFG is an important region for executive functions in dual-task performance (Rossi et al., 2009) and in switching attentional control on the basis of changing task demands (Szameitat et al., 2002). When it comes to text reading, the modulation from the MFG to the dorsal and ventral connections may be corresponding to the coordination between sub-processes of text reading. In contrast, there was no reliable bottom–up effect except for a negative EC from the left IPS to the left MFG at rest. We believe that text reading is a more volitional task relative to single character reading, so it is more likely to be dominated by a top–down control.

The results of FC analyses are largely in line with those of EC analyses, which supported the existence of the cooperation among regions in the dorsal-ventral visual network for text reading. Based on Zhou et al. (2015), the present study has used a dual-route approach to investigate the dorsal-ventral FCs in reading-driven fMRI and rs-fMRI. The current results of FC analyses in rs-fMRI for adults are generally similar to our previous rs-fMRI study for children (i.e., Zhou et al., 2015): the FC strength of MFG-IPS was positively correlated with the scores of reading fluency but not lexical decision, suggesting the unique contribution of the dorsal visual system to text reading abilities. Additionally, the FC analyses in reading-driven fMRI have further implicated the importance of this dorsal-ventral visual network for on-line text reading. Interestingly, we observed a negative correlation between the FC strength of VWFA-IPS and the score of lexical decision. This result may indicate that word processing in the VWFA needs to be supplemented by the deployment of attention in the IPS, especially for individuals with poor ability of lexical processing.

In general, our analyses suggest that the brain connectivity patterns in reading-driven fMRI and rs-fMRI are consistent with each other. Previous research has revealed that there are common mechanisms for reading-driven fMRI and rs-fMRI (Mennes et al., 2010). In reading research, Schurz et al. (2014b) explored the FCs in both reading-driven fMRI (tasks: visual word reading and phonological lexical decision) and rs-fMRI in developmental dyslexia, and found that there were consistent patterns in these two modalities. The present study has found that the FCs and the ECs for MFG-IPS and IPS-VWFA coupling were quite reliable in reading-driven fMRI and in rs-fMRI. As the resting-state connectivity is assumed to reflect a history of consistent and repeated co-activations of areas (Dosenbach et al., 2007; Fair et al., 2007), the identified stable connections are possibly due to more fundamental cognitive function such as visual search, a foundational process for text reading. However, we have observed the disconnected FC and EC between the MFG and the VWFA in resting states. Mennes et al. (2013) have proposed that some areas may show weak correspondence between the brain’s intrinsic and extrinsic functional architectures, and these areas are typically those that display more adaptive and context-dependent functional interactions. VWFA may be one such region, as previous studies have also indicated that the activation of VWFA is likely influenced by context-dependent higher-level processing (Dehaene and Cohen, 2011; Price, 2012). Thus, the magnitude of attentional modulation of VWFA activity in text reading may explain the inconsistent results between resting-state versus reading-driven fMRI patterns in the current study. In other words, during attention-demanding tasks of text reading, the VWFA-MFG ventral pathway is more likely to be activated in order to convert visual input to high-level word-to-text integration, whereas in a resting-state, this ventral pathway is less likely to become active. Notably, as we used datasets from different participants in Experiments 1 and 2, the consistency in brain connection patterns between reading-driven fMRI and rs-fMRI was consolidated whereas their difference should be interpreted with caution.

While the FC reveals non-directional relationships between brain regions, the EC indicates a directional influences from one region to another. However, as there has been little research using both FC and EC in the same analyses, the specific relationship between FC and EC effects is somewhat difficult to explain. At the very least, these two effects are not independent of each other. For example, the correlation (i.e., FC) between two areas can be caused by a common input (i.e., EC) of another area from past fluctuations (Friston, 2011). Consistently, we have observed concurrent and similar pattern of FC and EC networks in the present study, suggesting a complex relationship between FC and EC effects. In addition, while we have successfully discovered the brain–behavior correlations for FC at rest (i.e., correlation between MFG-IPS connectivity and reading fluency), there is no evidence for the relationship between EC and the reading performance scores. We suspect that EC is more dynamic (activity-dependent) relative to FC, so the brain–behavior relationship is relatively difficult to detect at rest. Such discrepancies between FC and EC patterns and the corresponding effects remain to be investigated in future research.

Although, we have identified the important role of the dorsal-ventral visual network in text reading, the nature of this network needs further clarification. For instance, is this network also related to general comprehension? Currently, we believe that the found connections are more associated with visual/attention aspects of reading relative to general comprehension, as typical regions for general language comprehension (e.g., the left IFG and MTG; Constable et al., 2004; Buchweitz et al., 2009) were not included in the dorsal-ventral visual system. However, these connections may be potentially modulated by comprehension processes. Future research is needed to directly compare the dorsal-ventral visual system in self-pace reading with comprehension processes (e.g., text reading) and without comprehension processes (e.g., word list reading). Another noticeable point is that the results of present study are based on text reading in the Chinese writing system. The Chinese text is written in a series of characters of roughly uniform height and width. Unlike alphabetic writing system, Chinese orthographic system is well-known for the complex visual form of characters and the lack of inter-word spaces for word segmentation in text. These properties were tightly associated with the function of dorsal visual regions such as MFG and IPS. However, Wang X.J. et al. (2015) have found that the difference in activation between Chinese and English story reading mainly situated in ventral visual regions. An interesting question for further research is whether the connectivity patterns of the dorsal-ventral visual system in Chinese can be generalized to alphabetic writing systems.

## Conclusion

Readers recruit both the dorsal and ventral visual pathways when reading a narrative text. Previous fMRI studies on text reading has identified a distributed set of frontal, parietal, and occipital-temporal regions within this dual-route system. The current study examined the interactions among these regions in text reading using FC and EC analyses in task-driven fMRI and rs-fMRI. Our analyses suggest a dynamic picture in which the dorsal and ventral systems involving MFG, IPS, and VWFA cooperate with each other and causally influence one another during natural text reading.

## Author Contributions

Study conception and design: HS, WZ; acquisition of data: XW, ZX; analysis and interpretation of data: WZ; drafting of manuscript: WZ, HS; critical revision: HS, PL, YB.

## Conflict of Interest Statement

The authors declare that the research was conducted in the absence of any commercial or financial relationships that could be construed as a potential conflict of interest.

## Footnotes

1 http://www.fiL.ion.ucL.ac.uk/spm

2 http://www.restfmri.net
