# Supplementary material for: Neural Mechanisms of Dorsal and Ventral Visual Regions during Text Reading
Source: Front Psychol. 2016 Sep 15;7:1399. doi: 10.3389/fpsyg.2016.01399 (PMC5023685; doi:10.3389/fpsyg.2016.01399)
Supplement: Supplementary file 1 [file Presentation_1.PPTX]

## Slide 1
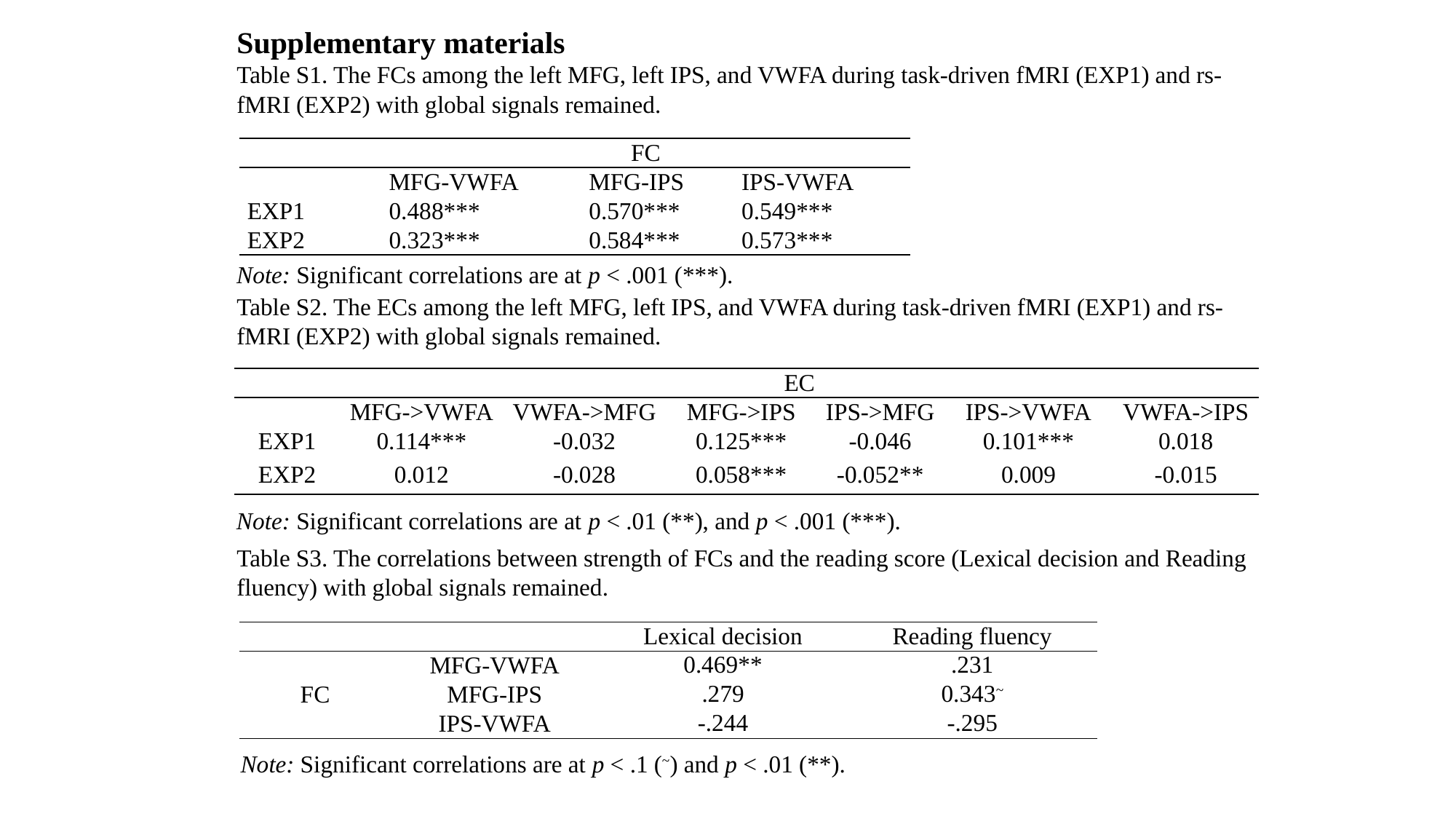

# Supplementary materials Table S1. The FCs among the left MFG, left IPS, and VWFA during task-driven fMRI (EXP1) and rs-fMRI (EXP2) with global signals remained.
| | FC | | |
| --- | --- | --- | --- |
| | MFG-VWFA | MFG-IPS | IPS-VWFA |
| EXP1 | 0.488\*\*\* | 0.570\*\*\* | 0.549\*\*\* |
| EXP2 | 0.323\*\*\* | 0.584\*\*\* | 0.573\*\*\* |
Table S2. The ECs among the left MFG, left IPS, and VWFA during task-driven fMRI (EXP1) and rs-fMRI (EXP2) with global signals remained.
Note: Significant correlations are at p < .001 (***).
| | EC | | | | | |
| --- | --- | --- | --- | --- | --- | --- |
| | MFG->VWFA | VWFA->MFG | MFG->IPS | IPS->MFG | IPS->VWFA | VWFA->IPS |
| EXP1 | 0.114\*\*\* | -0.032 | 0.125\*\*\* | -0.046 | 0.101\*\*\* | 0.018 |
| EXP2 | 0.012 | -0.028 | 0.058\*\*\* | -0.052\*\* | 0.009 | -0.015 |
Table S3. The correlations between strength of FCs and the reading score (Lexical decision and Reading fluency) with global signals remained.
Note: Significant correlations are at p < .01 (**), and p < .001 (***).
| | | Lexical decision | Reading fluency |
| --- | --- | --- | --- |
| FC | MFG-VWFA | 0.469\*\* | .231 |
| | MFG-IPS | .279 | 0.343~ |
| | IPS-VWFA | -.244 | -.295 |
Note: Significant correlations are at p < .1 (~) and p < .01 (**).

## Slide 2
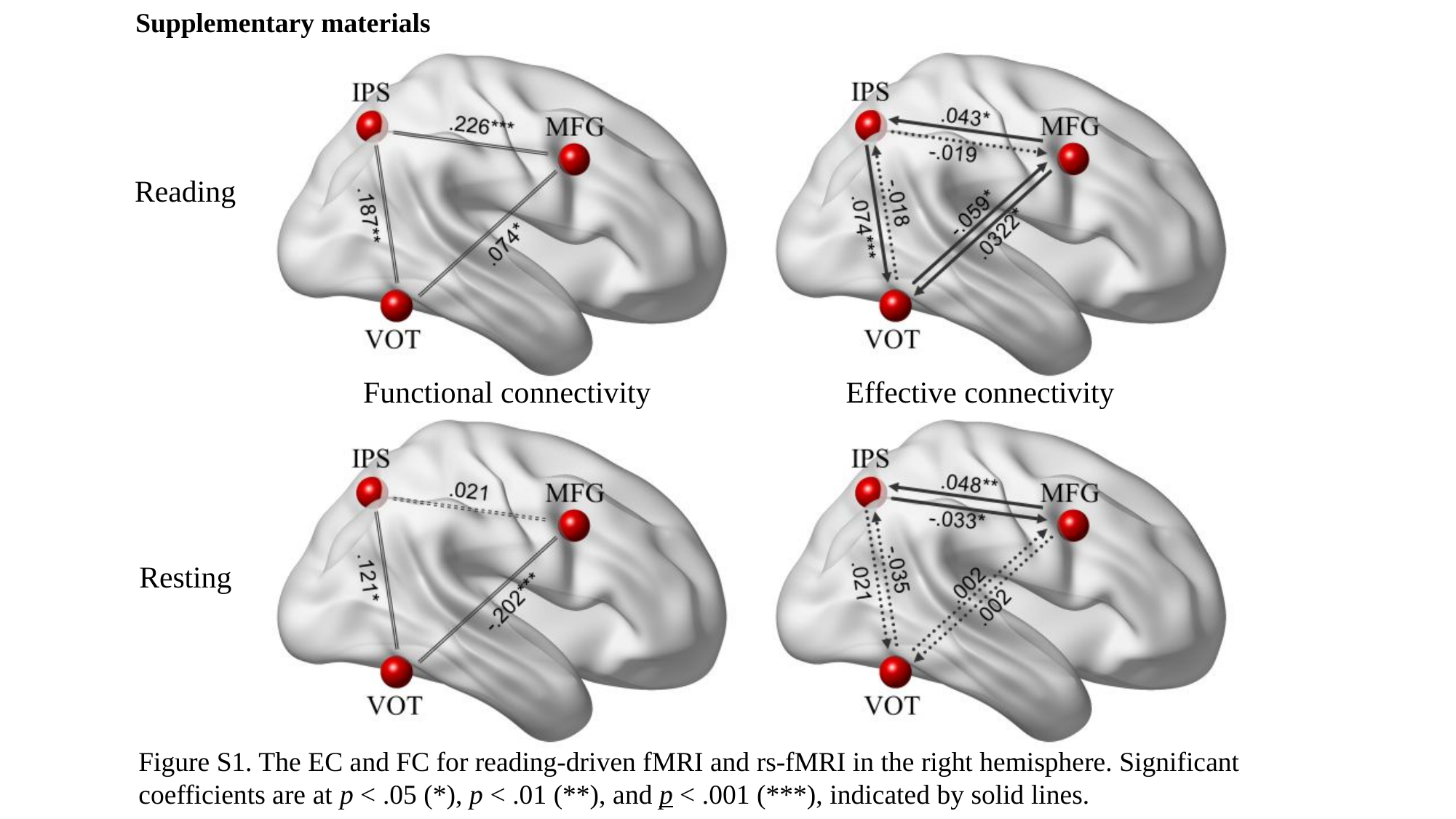

Supplementary materials
Reading
Functional connectivity
Effective connectivity
Resting
Figure S1. The EC and FC for reading-driven fMRI and rs-fMRI in the right hemisphere. Significant coefficients are at p < .05 (*), p < .01 (**), and p < .001 (***), indicated by solid lines.
